# Clinical Study of Sintilimab as Second-Line or Above Therapy in Patients With Advanced or Metastatic Gastric Cancer: A Retrospective Study

**DOI:** 10.3389/fonc.2021.741865

**Published:** 2021-09-22

**Authors:** Caiyun Nie, Huifang Lv, Yingjun Liu, Beibei Chen, Weifeng Xu, Jianzheng Wang, Xiaobing Chen

**Affiliations:** ^1^ Department of Medical Oncology, Affiliated Cancer Hospital of Zhengzhou University, Henan Cancer Hospital, Zhengzhou, China; ^2^ State Key Laboratory of Esophageal Cancer Prevention & Treatment, Zhengzhou University, Zhengzhou, China; ^3^ Department of General Surgery, Affiliated Cancer Hospital of Zhengzhou University, Henan Cancer Hospital, Zhengzhou, China

**Keywords:** gastric/gastroesophageal junction adenocarcinoma, sintilimab, immunotherapy, efficacy, Lauren classification

## Abstract

**Background:**

The present study was conducted to analyze the clinical efficacy and safety of sintilimab as second-line or above therapy for patients with advanced or metastatic gastric cancer.

**Methods:**

Patients with advanced or metastatic gastric cancer that progressed after prior systemic therapies and treated with sintilimab from March 2019 to July 2020 were retrospectively analyzed in this study. The primary end point was progression-free survival (PFS). Secondary end points included objective response rate (ORR), disease control rate (DCR), overall survival (OS), and safety.

**Results:**

Fifty-two patients with advanced or metastatic gastric cancer received sintilimab monotherapy or combination therapy after they failed from prior systemic therapies. Eight patients achieved partial response (PR), 26 patients had stable disease (SD), and 18 patients had progressive disease (PD). The ORR and DCR were 15.4% (8/52) and 65.4% (34/52), respectively. Median PFS was 2.5 months (95% CI = 2.0–3.0), and median OS was 5.8 months (95% CI = 4.9–6.7). The ORR and DCR were 30.0% (6/20) and 80.0% (16/20), respectively, in intestinal subtype, which were superior than in non-intestinal subtype (ORR: 6.3%, DCR: 56.3%). Patients with intestinal subtype obtained longer PFS (4.0 *vs.* 1.9) and OS (9.0 *vs.* 4.1) than those with non-intestinal subtype. The incidence of grade 3–4 adverse events was 44.2%.

**Conclusions:**

Sintilimab monotherapy or combination therapy provides a feasible therapeutic strategy for patients with advanced or metastatic gastric cancer who failed from prior systemic therapies. The efficacy of sintilimab in intestinal subtype was superior than that in non-intestinal subtype.

## Introduction

Most cases of gastric cancer are advanced at diagnosis, the prognosis is extremely poor, and there is a lack of effective treatment. For some strictly selected cases, local treatment, including surgical resection and hyperthermic intraperitoneal chemotherapy, may be able to improve the prognosis of patients (1, 2). Medical treatment, including chemotherapy and targeted therapy, is currently the main treatment for advanced or metastatic gastric cancer. However, the efficacy of chemotherapy drugs seems to have reached a plateau, and the progress of traditional targeted therapy drugs is relatively slow (3, 4). As an emerging treatment method, immunotherapy is the current research hotspot, and it is hoped that it can further improve the curative effect of advanced gastric cancer (5). Based on the ATTRACTION-02 and KEYNOTE-059 studies, nivolumab and pembrolizumab have been approved in advanced gastric cancer in Japan and the United States as third-line treatment, respectively (6, 7).

Sintilimab is a fully human IgG4 monoclonal antibody that acts on PD-1 and its ligands. It is the second approved Chinese PD-1 inhibitor in China (8). In 2018, sintilimab received indications for relapsed/refractory Hodgkin’s lymphoma in China. Clinical trials on other tumor types are also underway, including lymphoma (9), non-small cell lung cancer (10), liver cancer (11), esophageal cancer (12), and gastric cancer (13). Compared with other PD-1 inhibitors, sintilimab has similar anti-tumor effects, better safety, and economic advantages. A phase IB study evaluating sintilimab combined with XELOX as first-line treatment for HER-2 negative gastric and gastroesophageal junction (GEJ) adenocarcinoma showed that the objective response rate (ORR) of sintilimab treatment was 85%, and the disease control rate (DCR) was 100% (13).

Although many advances have been made in immunotherapy of gastric cancer, there are still many problems. Arranging the troops, optimizing the treatment strategy, and better targeting the patients who will benefit from immunotherapy based on biomarkers have become an urgent clinical goal (14). Gastric cancer is highly heterogeneous. In the classical Lauren classification, gastric cancer can be divided into intestinal, diffuse, and mixed types. Previous studies have shown that immunotherapy is not effective in diffuse gastric cancer (6, 15). The present study was performed to evaluate the efficacy and safety of sintilimab for patients with advanced or metastatic gastric cancer as second-line or above therapy.

## Methods

### Patient Population

From March 2019 to July 2020, patients with advanced or metastatic gastric cancer who failed from prior systemic therapies at Henan Cancer Hospital were retrospectively analyzed. Eligibility criteria were as follows: 1) patients with gastric cancer that progressed after first-line chemotherapy and treated with sintilimab as second-line or above therapy; 2) Eastern Cooperative Oncology Group (ECOG) performance status 0/1; 3) measurable disease per Response Evaluation Criteria in Solid Tumors version 1.1 (RECIST v1.1), at least one lesion can be measured by imaging examination, and the lesion measured by spiral CT or MRI is ≥10 mm; and 4) adequate organ function.

### Study Treatment

Sintilimab was administered *via* intravenous infusion at a dose of 200 mg once every 3 weeks until disease progression, unacceptable toxicity, or death. In this study, sintilimab monotherapy and combination therapy were the two regimens. In the combination therapy regimen, sintilimab was given with concurrent chemotherapy or targeted therapy, including apatinib, trastuzumab, or nab-paclitaxel.

### Efficacy and Safety Assessments

The primary end point was progression-free survival (PFS). Secondary end points included ORR, DCR, overall survival (OS), and safety. After treatment, all patients underwent imaging examination every two cycles to evaluate the clinical efficacy. The efficacy evaluation criteria are RECIST version 1.1 response evaluation criteria in solid tumors, including complete response (CR), partial response (PR), stable disease (SD), and progressive disease (PD). The ORR was CR + PR, and the DCR was CR+ PR and SD. Adverse events (AEs) were assessed according to the Common Terminology Criteria for Adverse Events, version 4.0.

### Statistical Analysis

Survival curves were estimated using the Kaplan–Meier method and compared by log-rank test. PFS was defined as the period from the time of treatment with sintilimab to disease progression or patient death due to any cause. OS was defined as the period from the time of treatment with sintilimab to patient death from any cause or last follow-up. ORR and DCR with 95% CI were calculated using the exact method based on binomial distribution. Safety and efficacy were analyzed in all patients who received ≥1 dose of study treatment. Safety was analyzed using descriptive statistics. All the statistical and descriptive analyses were conducted using SPSS software version 17.0 (SPSS, Chicago, IL). *p* < 0.05 was considered significant.

## Results

### Patient and Treatment Characteristics

A total of 52 patients with advanced or metastatic gastric cancer who progressed after first-line treatment were retrospectively analyzed. **Table 1** summarizes patient and treatment characteristics. The median age was 64 years (range 30–80), with 17 female patients and 35 male patients. Twenty-three patients had advanced gastric cancer, and the other 29 patients had GEJ adenocarcinoma. All the patients were diagnosed as advanced or recurrent; the metastatic sites included the intra-abdominal lymph node (65.4%), liver (42.3%), peritoneum (28.8%), and lung (25%). In this study, 19 patients (36.5%) received sintilimab as second-line treatment and the other 33 patients (63.5%) as third or above treatment. Eight patients received sintilimab as monotherapy, and 44 patients received sintilimab combination therapy. In the 44 patients who received sintilimab combination therapy, 24 patients received sintilimab combined with apatinib, and the other 20 patients received sintilimab combined with nab-paclitaxel or irinotecan. In the early days, due to the limitation of testing reagents, PD-L1 was not a routine test item in the pathology department of our center. Among the 52 patients in this study, there were 21 patients with PD-L1 expression results, of which seven were PD-L1 positive and 14 were PD-L1 negative.

**Table 1:** Patient and treatment characteristics.

Characteristic	Total (n = 52) n (%)	Monotherapy (n = 8) n (%)	Combination therapy (n = 44) n (%)
**Age (years, median)**	64 (30–80)	67.5 (62–75)	61 (30–80)
**Gender**			
Female	17 (32.7%)	4 (50%)	13 (29.5%)
Male	35 (67.3%)	4 (50%)	31 (70.5%)
**ECOG**			
0–1	40 (76.9%)	5 (62.5%)	35 (79.5%)
2	12 (23.1%)	3 (37.5%)	9 (20.5%)
**Primary tumor site**			
Gastric	23 (44.2%)	0 (0%)	23 (52.3%)
GEJ	29 (55.8%)	8 (100%)	21 (47.7%)
**Histopathology**			
Intestinal	20 (38.5%)	1 (12.5%)	19 (43.2%)
Diffuse	22 (42.3%)	6 (75%)	16 (36.4%)
Mixed	10 (19.2%)	1 (12.5%)	9 (20.4%)
**MSI**			
dMMR	1 (1.9%)	0 (0%)	1 (2.3%)
MSS	51 (98.1%)	8 (100%)	43 (97.7%)
**Metastatic site**			
Lymph node	34 (65.4%)	5 (62.5%)	29 (65.9%)
Peritoneum	15 (28.8%)	3 (37.5%)	12 (27.3%)
Liver	22 (42.3%)	3 (37.5%)	19 (43.2%)
Lung	13 (25%)	4 (50%)	9 (20.5%)
Others	9 (17.3%)	0 (%)	9 (20.5%)
**Number of metastatic sites**			
**1–2**	41 (78.8%)	6 (75%)	35 (79.5%)
**≥3**	11 (21.2%)	2 (25%)	9 (20.5%)
**Treatment line**			
**2**	19 (36.5%)	2 (25%)	17 (38.6%)
**3–5**	33 (63.5%)	6 (75%)	27 (61.4%)

ECOG, Eastern Cooperative Oncology Group performance status; GEJ, gastroesophageal junction tumors; MSI, microsatellite instability; dMMR, deficient mismatch repair; MSS, microsatellite stability.

### Efficacy

In the general population, CR was not observed, eight patients achieved PR, 26 patients had SD, and 18 patients had PD. The ORR and DCR were 15.4% (8/52) and 65.4% (34/52), respectively. In the intestinal subtype population, six patients achieved PR, 10 patients had SD, and four patients had PD; the ORR and DCR were 30.0% (6/20) and 80.0% (16/20), respectively. In the non-intestinal subtype population, two patients achieved PR, 16 patients had SD, and 14 patients had PD; the ORR and DCR were 6.3% (2/32) and 56.3% (18/32), respectively. In the PD-L1-positive population, four patients achieved PR, three patients had SD, and no patients had PD; the ORR and DCR were 57.1% (4/7) and 100% (7/7), respectively. In the PD-L1-negative population, one patient achieved PR, eight patients had SD, and five patients had PD; the ORR and DCR were 7.1% (1/14) and 64.3% (9/14), respectively. In the sintilimab monotherapy population, one patient achieved PR, three patients had SD, and four patients had PD; the ORR and DCR were 12.5% (1/8) and 50.0% (4/8), respectively. In the combination therapy population, seven patients achieved PR, 23 patients had SD, and 14 patients had PD; the ORR and DCR were 15.9% (7/44) and 68.2% (30/44), respectively (**Table 2**).

**Table 2:** Efficacy of sintilimab in patients with advanced or metastatic gastric cancer.

Parameter	Best response				ORR	DCR	Median PFS (95% CI)	Median OS (95% CI)
	CR	PR	SD	PD				
Total	0	8	26	18	15.4% (8/52)	65.4% (34/52)	2.5 (2.0–3.0)	5.8 (4.9–6.7)
Lauren classification								
Intestinal	0	6	10	4	30.0% (6/20)	80.0% (16/20)	4.0 (3.1–4.8)	9.0 (6.7–11.3)
Non-intestinal	0	2	16	14	6.3% (2/32)	56.3% (18/32)	1.9 (1.2–2.6)	4.1 (2.7–5.4)
Treatment programs								
Monotherapy	0	1	3	4	12.5% (1/8)	50.0% (4/8)	1.5 (0.3–2.7)	4.0 (0–8.7)
Combination	0	7	23	14	15.9% (7/44)	68.2% (30/44)	2.9 (2.3–3.5)	6.0 (5.0–7.0)
PD-L1								
Positive	0	4	3	0	57.1% (4/7)	100.0% (7/7)	5.0 (4.0–6.0)	12.1 (6.4–17.8)
Negative	0	1	8	5	7.1% (1/14)	64.3% (9/14)	2.0 (1.1–2.9)	4.1 (2.0–6.2)
Combination type								
Apatinib	0	3	13	8	12.5% (3/24)	66.7% (16/24)	2.4 (1.7–3.1)	6.0 (2.1–9.9)
Chemotherapy	0	4	10	6	20.0% (4/20)	70.0% (14/20)	2.9 (1.9–3.9)	5.8 (4.7–6.9)

CR, complete response; PR, partial response; SD, stable disease; PD, progressive disease; ORR, overall response rate; DCR, disease control rate; PFS, progression-free survival; OS, overall survival.

**Figure 1:**
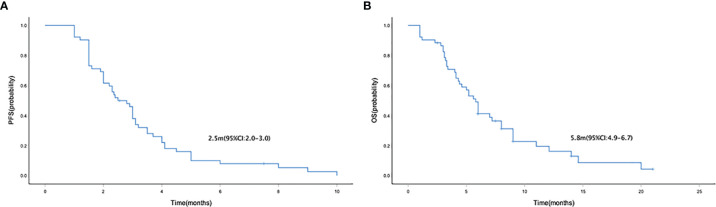
Kaplan–Meier curve of PFS **(A)** and OS **(B)** in the general population. PFS, progression-free survival; OS, overall survival.

**Figure 2:**
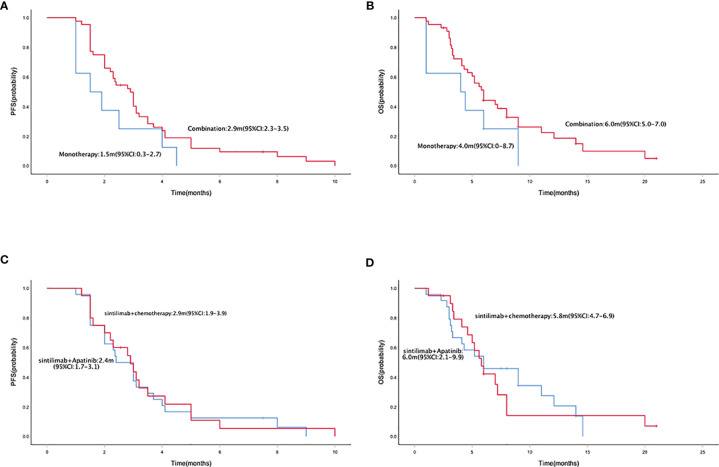
Kaplan–Meier curve of PFS **(A)** and OS **(B)** in sintilimab monotherapy and combination therapy population. Kaplan–Meier curve of PFS **(C)** and OS **(D)** in sintilimab combination therapy population. PFS, progression-free survival; OS, overall survival.

**Figure 3:**
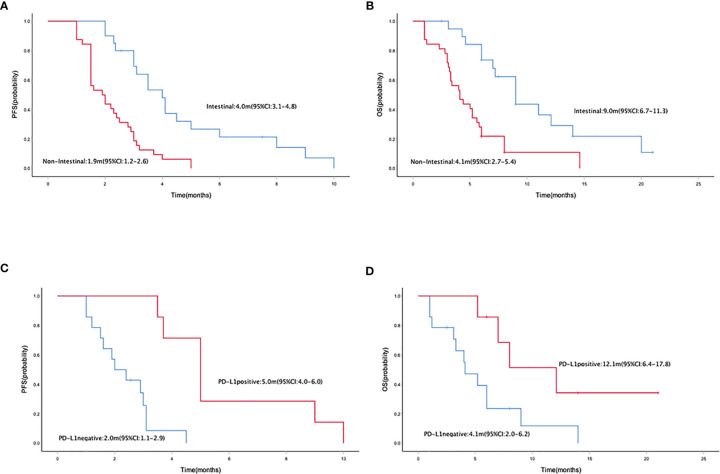
Kaplan–Meier curve of PFS **(A)** and OS **(B)** in intestinal and non-intestinal subtype populations. Kaplan–Meier curve of PFS **(C)** and OS **(D)** in PD-L1-positive and PD-L1-negative populations. PFS, progression-free survival; OS, overall survival.

Median PFS and OS were 2.5 months (95% CI = 2.0–3.0) (**Figure 1A**) and 5.8 (95% CI = 4.9–6.7) months (**Figure 1B**), respectively. The median PFS in patients who received mono- and combo-regimens was 1.5 (95% CI = 0.3–2.7) and 2.9 (95% CI = 2.3–3.5) months, respectively (*p* = 0.088) (**Figure 2A**); and OS was 4.0 (95% CI = 0–8.7) and 6.0 (95% CI = 5.0–7.0) months, respectively (*p* = 0.133) (**Figure 2B**). Twenty-four patients who received sintilimab combined with apatinib obtained 2.4 (95% CI = 1.7–3.1) months’ PFS and 6.0 (95% CI = 2.1–9.9) months’ OS. Twenty patients who received sintilimab combined with nab-paclitaxel or irinotecan obtained 2.9 (95% CI = 1.9–3.9) months’ PFS and 5.8 (95% CI = 4.7–6.9) months’ OS (for PFS, *p* = 0.818; for OS, *p* = 0.883) (**Figures 2C, D**). For Lauren classification, the median PFS in intestinal and non-intestinal subtypes was 4.0 (95% CI = 3.1–4.8) months and 1.9 (95% CI = 1.2–2.6) months, respectively (*p* = 0.000) (**Figure 3A**). The median OS in intestinal and non-intestinal subtypes was 9.0 (95% CI = 6.7–11.3) months and 4.1 (95% CI = 2.7–5.4) months, respectively (*p* = 0.000) (**Figure 3B**). The median PFS in PD-L1-positive and PD-L1-negative patients was 5.0 (95% CI = 4.0–6.0) months and 2.0 (95% CI = 1.1–2.9) months, respectively (*p* = 0.000) (**Figure 3C**). The median OS in PD-L1-positive and PD-L1-negative patients was 12.1 (95% CI = 6.4–17.8) months and 4.1 (95% CI = 2.0–6.2) months, respectively (*p* = 0.027) (**Figure 3D**).

### Safety

In terms of safety, all of the 52 patients reported at least one treatment-related AE (TRAE). In general, sintilimab treatment was well tolerated, and only two patients discontinued treatment due to intolerable toxicity. Most of the AEs were grade 1–2 (**Table 3**). Grade 3–4 adverse reactions occurred in 23 (44.2%) patients. No unexpected side effects or treatment-related death were observed. The most common sintilimab-related AEs were hematological toxicity, including anemia (n = 32, 61.5%), decreased neutrophil count (n = 42, 80.8%), decreased platelet (n = 31, 59.6%), and decreased white blood count (n = 41, 78.9%). Other common sintilimab-related AEs were pyrexia (n = 15, 28.8%), increased aspartate aminotransferase and alanine aminotransferase (n = 16, 30.8%), hypothyroidism (n = 12, 23.1%), rash (n = 18, 34.6%), and pneumonitis (n = 7, 13.5%). Grade 3–4 AE rash occurred in two patients. Apatinib-related AEs were secondary hypertension (n = 11, 21.2%), hand and foot syndrome (n = 8, 15.4%), and proteinuria (n = 6, 11.5%).

**Table 3:** Treatment-related adverse events (TRAEs).

Adverse event	All grade n (%)	≥Grade 3 n (%)
Any event	52 (100.0)	23 (44.2)
AE led to any treatment discontinuation	4 (7.7)	3 (5.8)
AE led to death	0 (0.0)	0 (0.0)
Event occurring in **≥**10% patients		
Pyrexia	15 (28.8)	0 (0.0)
Anemia	32 (61.5)	4 (7.7)
Decreased neutrophil count	42 (80.8)	8 (15.4)
Decreased platelet	31 (59.6)	12 (23.1)
Decreased white blood count	41 (78.9)	6 (11.5)
Fatigue	20 (38.5)	0 (0.0)
Nausea	15 (28.8)	0 (0.0)
Vomiting	8 (15.4)	0 (0.0)
Increased aspartate aminotransferase	16 (30.8)	0 (0.0)
Increased alanine aminotransferase	16 (30.8)	0 (0.0)
Proteinuria	6 (11.5)	0 (0.0)
Hypothyroidism	12 (23.1)	0 (0.0)
Hand and foot syndrome	8 (15.4)	2 (3.8)
Rash	18 (34.6)	2 (3.8)
Pneumonitis	7 (13.5)	0 (0.0)
Sensory neurotoxicity	10 (19.2)	1 (1.9)
Diarrhea	7 (13.5)	0 (0.0)
Secondary hypertension	11 (21.2)	3 (5.8)

AE, adverse event.

## Discussion

In the present study, the results from this retrospective study demonstrated favorable anti-tumor activity and manageable safety of sintilimab as second-line or above therapy for advanced or metastatic gastric cancer.

Immune checkpoint inhibitors have been approved in gastric cancer worldwide as a third-line treatment option. The results of the ATTRACTION-02 study in the Asian population showed that nivolumab treatment significantly reduced the risk of death of advanced or metastatic gastric cancer patients (6). The 1-year OS rates reached 26.2%. The National Medical Products Administration has approved the use of nivolumab in the treatment of patients with advanced or metastatic gastric cancer or GEJ adenocarcinoma who failed from two or more systemic treatment regimens. In the KEYNOTE-059 study, pembrolizumab was confirmed to be effective in the treatment of advanced or metastatic gastric cancer (7). But unfortunately, in the subsequent clinical trials where immunotherapy was moved to the second line before the treatment of advanced gastric cancer, the results of KEYNOTE-061 brought confusion to clinicians (15). The role of immune checkpoint inhibitors in the second-line treatment of advanced gastric cancer has not been established. However, data on second-line immunotherapy for gastric cancer are also accumulating. In this study, 19 patients (36.5%) received sintilimab as second-line treatment and the other 33 patients (63.5%) as third or above treatment. Whether in second-line or third to fifth-line treatment, sintilimab demonstrated encouraging results. The results of this present study substantiate evidence for gastric cancer immunotherapy, especially in the second-line immunotherapy of gastric cancer.

The optimal drug treatment model of immunotherapy for gastric cancer is still inconclusive. In this study, eight patients received sintilimab monotherapy, and another 44 patients received sintilimab combination therapy. In terms of efficacy, the ORR and DCR of the combined treatment group were higher than those of the monotherapy group, and the PFS and OS were also superior. For drug safety, sintilimab monotherapy had a lower incidence of TRAEs and superior tolerability. Most patients with gastric cancer cannot tolerate chemotherapy for a long time because of disease progression. For patients with poor ECOG scores, immunotherapy like sintilimab monotherapy could an optional strategy in terms of safety profile.

In this study, 24 patients received sintilimab combined with apatinib, and the other 20 patients received sintilimab combined with nab-paclitaxel or irinotecan. In terms of efficacy, no significant difference was found. Currently, clinical trials are exploring the combination manner of immunotherapy. In addition to the traditional immunotherapy combined with chemotherapy, immunotherapy combined with targeted therapy has been proved to be an effective combination mode. HER-2 and VEGF are two vital targets (16, 17). Trastuzumab combined with pembrolizumab has achieved good results in patients with HER2-positive second-line and above treatment of gastric cancer. In this study, sintilimab combined with apatinib achieved significant efficacy. Studies had shown that antiangiogenic drugs can change the tumor immune microenvironment and enhance the efficacy of immunotherapy, which has become a new therapeutic strategy (18–20).

There are still no effective biomarkers to predict the efficacy of immunotherapy for gastric cancer. Some studies suggest that PD-L1 expression level, tumor mutational burden (TMB), Epstein–Barr virus (EBV) positive, and POLE gene mutation may be potential biomarkers to predict the efficacy of immunotherapy, but it has not been proved to be specific and effective enough, which is still controversial (21). At present, deficient mismatch repair/microsatellite instability-high (dMMR/MSI-H) is the only effective marker for anti-PD-1 treatment (22, 23). However, in gastric cancer, MSI-H accounted for only 20%, and 80% of gastric cancer patients had microsatellite stability (MSS) (24). In this study, only one patient was diagnosed as dMMR, and the others were MSS. In the 21 patients with PD-L1 expression results, PD-L1-positive patients exhibited better anti-tumor immune response and longer PFS and OS. Since only some patients have PD-L1 results, the predictive value of PD-L1 in the immunotherapy of sintilimab for gastric cancer still needs to be further explored.

Previous studies suggested that the expression level of PD-L1 in diffuse gastric cancer may be lower than that in intestinal type. ONO-4538 study and KEYNOTE-061 study demonstrated that immunotherapy with nivolumab or pembrolizumab was not effective in diffuse gastric cancer. Our present study showed the ORR and DCR in the intestinal subtype population were significantly higher than in the non-intestinal subtype population; meanwhile, the intestinal subtype gastric cancer population has achieved better prognosis. The relationship between the efficacy of sintilimab immunotherapy and Lauren classification has not been reported. Our research suggests that the Lauren classification may affect the effect of sintilimab immunotherapy.

For safety, sintilimab treatment was well tolerated, and only two patients discontinued treatment due to intolerable toxicity. The most common sintilimab-related AEs were consistent with previous studies (25, 26). Among all levels of AEs, hematological toxicity is most common, including decreased neutrophil count, decreased white blood count, decreased platelet, and anemia. Rash was one of the most frequent grade 3 or 4 AEs. Other ≥grade 3 AEs were hand and foot syndrome, sensory neurotoxicity, and secondary hypertension, which were similar to previous data of chemotherapy and apatinib treatment.

A retrospective study obtained from a single center with not sufficiently large patient cases is the limitation of our study. Thus, we should design and conduct large randomized and prospective trials to confirm the clinical value of sintilimab monotherapy or combination therapy in advanced or metastatic gastric cancer.

## Conclusion

Sintilimab monotherapy or combination therapy provides a feasible therapeutic strategy in patients with advanced or metastatic gastric cancer who progressed after prior systemic therapies, and a median PFS of 2.5 months was obtained with well-tolerated toxicity. The efficacy of sintilimab in intestinal subtype was superior than in non-intestinal subtype.

## Data Availability Statement

The raw data supporting the conclusions of this article will be made available by the authors, without undue reservation.

## Ethics Statement

The studies involving human participants were reviewed and approved by the ethics committee of the Affiliated Cancer Hospital of Zhengzhou University. The patients/participants provided their written informed consent to participate in this study.

## Author Contributions

CN and XC designed the research, analyzed the data, and drafted the paper. CN, HL, YL, BC, and WX were mainly responsible for data collection and analysis. CN, XC, and JW were primarily responsible for statistical analysis. CN, HL, and XC contributed to the study design and revised the manuscript. All authors contributed to the article and approved the submitted version.

## Funding

This work was supported by the National Natural Science Foundation of China (No. 81472714), Medical Science and Technique Foundation of Henan Province (No. 212102310623) and 1000 Talents Program of Central plains (No. 204200510023).

## Conflict of Interest

The authors declare that the research was conducted in the absence of any commercial or financial relationships that could be construed as a potential conflict of interest.

## Publisher’s Note

All claims expressed in this article are solely those of the authors and do not necessarily represent those of their affiliated organizations, or those of the publisher, the editors and the reviewers. Any product that may be evaluated in this article, or claim that may be made by its manufacturer, is not guaranteed or endorsed by the publisher.
